# Morphological and Genetic Structure of Two Equivalent* Astyanax* Species (Characiformes: Characidae) in the Region of Paranaíba Arc

**DOI:** 10.1155/2019/6507954

**Published:** 2019-04-17

**Authors:** Renan Rodrigues Rocha, Rosana de Mesquita Alves, Rubens Pasa, Karine Frehner Kavalco

**Affiliations:** ^1^Laboratory of Ecological and Evolutionary Genetics, Institute of Biological and Health Sciences, Federal University of Viçosa, Campus Rio Paranaíba, BR 354, Km 310, Campus Universitário, Post Code: 38810-000, Rio Paranaíba, MG, Brazil; ^2^Graduate Program in Management and Conservation of Natural and Agricultural Ecosystems, Federal University of Viçosa, Campus Florestal, Rodovia LMG 818, Km 06 Post Code: 35690-000, Florestal, MG, Brazil

## Abstract

The* Astyanax scabripinnis* complex is composed of a large number of almost morphological indistinguishable species, including* Astyanax paranae* and* Astyanax rivularis*, which exist in the Paraná and São Francisco Basins, respectively, and sometimes are considered subspecies of the* A. scabripinnis* group or even are cited just as* A. scabripinnis*. The two river basins are separated by the Upper Paranaíba Arc, likely the main cause of the isolation of these species. We used geometric morphometric tools and DNA analyses of populations of both species to identify the differences between them. Geometric morphometrics separated the two species into distinct groups, whose main difference was the body depth. This is generally related to the speed of the water flow in the river basins. The maximum likelihood phylogram based on mitochondrial DNA sequences formed two main clades: one composed of the population of* A. rivularis* and the other, of* A. paranae*. In the haplotype network, the species were similarly separated into two groups from the same ancestral haplotype, with* A. rivularis* dispersing into two lineages in the São Francisco River Basin. The distribution of* A. paranae* is a consequence of a secondary dispersion event in the Paraná River Basin. It forms two lineages from a haplotype derived from the ancestor. The vicariant effect of separate basins, through the elevation of the Upper Paranaíba Arc, led to the allopatric speciation of the populations originating the present species. The results of geometric morphometrics and molecular data of the fish show the importance of this geological event in the biogeography and evolutionary history of the ichthyofauna of the region and indicate that the isolation of these species seems to be effective.

## 1. Introduction

The genus* Astyanax* Baird and Girard, 1854, is composed of fishes popularly known as piabas or lambaris. The genus belongs to the Characidae family and has about 160 species distributed on the Neotropical region watershed [1].

The species complex* Astyanax scabripinnis *is an example of the morphological and genetic diversity of the genus. Previously considered as a single species, Moreira Filho and Bertollo [2], proposed that it was a species complex, based on variations found in cytogenetic and phenotypic characteristics. Fishes of the complex usually inhabit the headwater of rivers and small streams [3], which results in isolated populations that could be driven to allopatric speciation [2].

The fishes of the complex are widely distributed through large hydrographic basins, as Paraná River basin and São Francisco River basin [4]. These two hydrographic basins were separated by the uplift of the Upper Paranaíba Arc, which increased the degree of isolation of the existent populations [5]. As a representative species from São Francisco River basin, we have* A. rivularis*; meanwhile from Upper Paraná river basin* A. paranae *can be found, with low tendency to migration [6].* Astyanax rivularis* and* A. paranae* are strongly related [7] and in spite of belonging to different basins these two species do have several ecological, morphological, and genetic similarities.

Delimiting related species or species within a complex is a hard task that demands studies on several areas to achieve a consensus on the procedure to separate them [8]. Despite the attempt to revise the* Astyanax scabripinnis* group, the taxonomists have difficulties to determine the nominal identification by just analyzing the samples. Molecular and morphological analyses could be some of the tools used for this delimitation [9].

Geometric morphometrics studies are efficient to demonstrate morphological differences between species within a species complex [10, 11], while phylogenetic and phylogeographic analyses based on mitochondrial DNA (mtDNA) allow elucidating evolutionary relationships and divergence of organisms [9].

*Astyanax paranae* and* A. rivularis* represent equivalent species formerly united under the* A. scabripinnis *complex that are distributed along an important watershed separating two major hydrographic basins. The present work aims to evaluate the morphological and/or genetic structuring among and within populations in adjacent region of the distribution of these two species.

## 2. Material and Methods

### 2.1. Specimens Sampling

**Figure 1:**
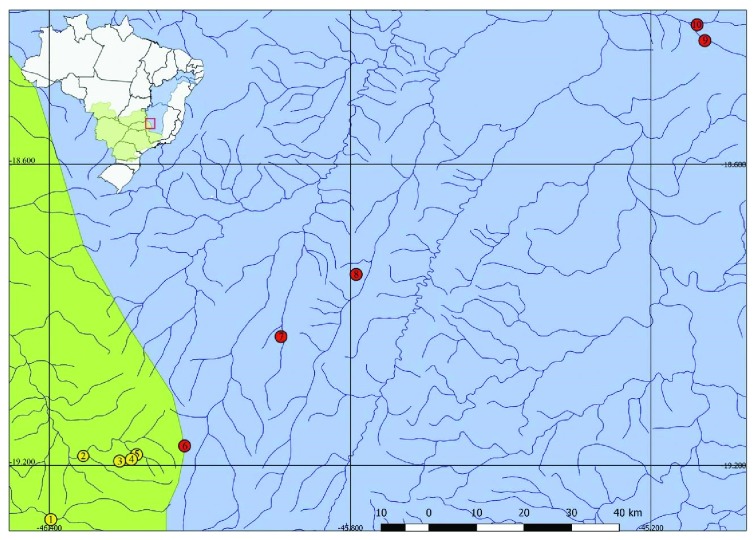
Sampling sites of specimens of this study. The locations numbered from 1 to 5 belong to the Paranaíba River basin; locations 6 to 10 belong to São Francisco River Basin. 1: São João river; 2: Paranaíba River; 3: Lava Pés stream; 4: Parque de Exposições stream; 5: Água Grande stream; 6: Abaeté river; 7: Tiros stream; 8; Borrachudo river; 9: Boi river; 10: Vereda Grande stream.

The individuals of* A. paranae* were collected in Paranaíba and São João rivers and Água Grande and Lava Pés streams, belonging to Upper Paraná river basin.* Astyanax rivularis* samples were collected from Do Boi, Borrachudo, and Abaeté rivers, and Tiros and Vereda Grande streams, along the region of Upper São Francisco (Figure 1). All the specimens were deposited in the collection of Laboratory of Ecological and Evolutionary Genetics at the Federal University of Viçosa (UFV),* campus* Rio Paranaíba. The sampling of the specimens was carried out in accordance with SISBIO, Sistema de Autorização e Informação em Biodiversidade (license number 1938128), and SISGEN, Sistema Nacional de Gestão do Patrimônio Genético e do Conhecimento Tradicional Associado (license number A9FE946). The euthanizing was carried out according to the recommendations of the Conselho Nacional de Controle de Experimentação Animal of Brazil (CONCEA).

### 2.2. Geometric Morphometrics

**Figure 2:**
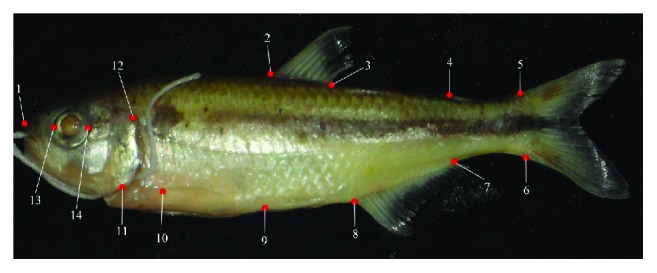
Analyzed landmarks: 1: rostrum; 2: anterior insertion; 3: posterior insertion of dorsal fin; 4: posterior insertion of adipose fin; 5: superior insertion of the first ray of caudal fin; 6: inferior insertion of final ray of caudal ray; 7: posterior insertion; 8:anterior insertion of anal fin; 9: insertion of ventral fin; 10: insertion of pectoral fin; 11: inferior limit; 12: superior limit of operculum; 13: anterior limit; 14: posterior limit of ocular orbit.

Forty-four individuals of* A. paranae* and forty-eight of* A. rivularis *were used for morphometrics analysis. The specimens were photographed with the use of a Sony Cyber-Shot camera, 14.1-megapixel resolution and 4x zoom. The software TPSUtil 1.6 [12] was used to group and format the data at a suitable file. Fourteen anatomic landmarks were selected to represent the general body shape of the fishes (Figure 2).

The landmarks were digitized with the use of the TpsDig 2.26 software system [13]. The transformation of data from the matrix by procrustes superposition was conducted with the aid of the PAST v2.17 software system [14], aiming to delete errors of scale, orientation, and position. The differences observed resulted only from shape variation [15, 16]. The difference between body shapes over species was determined by analyzing the canonical variables associated with Multivariate Analysis of Variance/Canonical Variance Analysis (MANOVA/CVA) [17], with the inference of consensus shape for each species on software MorphoJ 1.18 [18].

### 2.3. Phylogenetic Analysis

Twenty* A. paranae* individuals from the three populations of Paranaíba River Basin (Paranaíba and São João rivers, and Água Grande stream) and twenty-four* A. rivularis* individuals from São Francisco River Basin (Boi, Borrachudo, and Abaeté rivers; Tiros and Vereda Grande streams) were used for molecular analysis.

DNA was isolated from liver and heart samples of each specimen following the commercial protocol of the PureLink Genomic DNA kit by Invitrogen. The samples were quantified by electrophoresis on 1% agarose gel and Low Mass DNA ladder by Invitrogen, with posterior dilution to the final concentration of 10 ng/*μ*L of DNA. The amplification of Cytochrome* b *gene was carried out using primers H16460 - 5'CGAYCTTCGGATTACAAGAC3' and GluDG.L - 5'TGACCTGAARAACCAYCGTT3' [19]. The mitochondrial DNA from at least 3 individuals from each population was amplified and sequenced resulting in a fragment with 670 bp. The amplified sequences from Lava-Pés and Parque de Exposição streams were shorter and of poor quality than the others used in this work and therefore were excluded from further analysis. The PCR was conducted on a thermocycler with 25*μ*L reaction tubes containing 2,5*μ*L PCR 10X buffer, 1*μ*L MgCl_2 _in 50mM, 1,5*μ*L 10mM dNTP mix, 1*μ*L of each primer, 0,2*μ*L Taq polymerase, 5*μ*L of DNA template, and 12,8*μ*L of ultrapure water. The thermocycler program started at an initial step of 94°C for 4 minutes, followed by 35 cycles of 15 seconds at 94°C, 30 seconds at 56°C, and 2 minutes at 72°C, with a final extension of 10 minutes at 72°C, and was kept at 4°C [20].

The sequences obtained were aligned with ClustalW v1.6 algorithm [21] at the MEGA v6.06 [22]. The evolutionary model calculated by the software was HKY+G, with which the maximum likelihood phylogram was generated with 1000 replications. The* p*-distance between the sequences of the two species and the subsequently groupings was also calculated. The MEGA v6.06 software system [22], DnaSP 5.10 [23], and Network v4.6 were used to build the Cytochrome* b *haplotype network according to Median Joining Algorithm [24].

## 3. Results

### 3.1. Geometric Morphometrics

**Figure 3:**
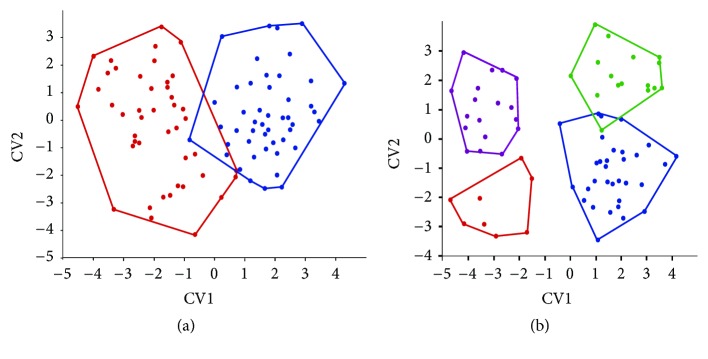
(a) Score position of MANOVA/CVA for each species, at the space of the first and second canonical axes. Red dots:* Astyanax paranae*, blue dots:* Astyanax rivularis*. (b) Score position of MANOVA/CVA for each lineage, at the space of the first and second canonical axes. Green dots: lineage A1, blue dots: lineage A2, purple dots: lineage B1, and red dots: lineage B2.

**Figure 4:**
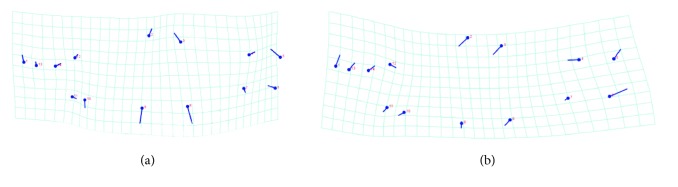
Consensus shape for each species. (a)* Astyanax rivularis*; (b)* Astyanax paranae*. The dot represents the landmarks and the trace of the direction where the landmarks are moving in the deformation grid.

The multivariate analysis (MANOVA/CVA) for the species showed shape differences between them (Wilke's lambda: 0.03236; df1 = 84; f = 4.69; p < 0.0001). The canonical axes CV1 explained 56.4% and CV2, 31% of the variation (Figure 3(a)). Alternatively, according to the molecular data, four groups were analyzed and showed morphological structuring (Wilke's lambda: 0.01105; df1 = 112; f = 4.01; p < 0.0001) with canonical axes CV1 explaining 60.1% and CV2, 22.1% of the variation (Figure 3(b)). The consensus shapes for each species are presented in Figure 4.* Astyanax paranae*'s shape shows a higher body depth, while* A. rivularis*' shape presented a lower body depth and longitudinal elongation, when compared to the first one.

### 3.2. Phylogenetic Analysis

**Figure 5:**
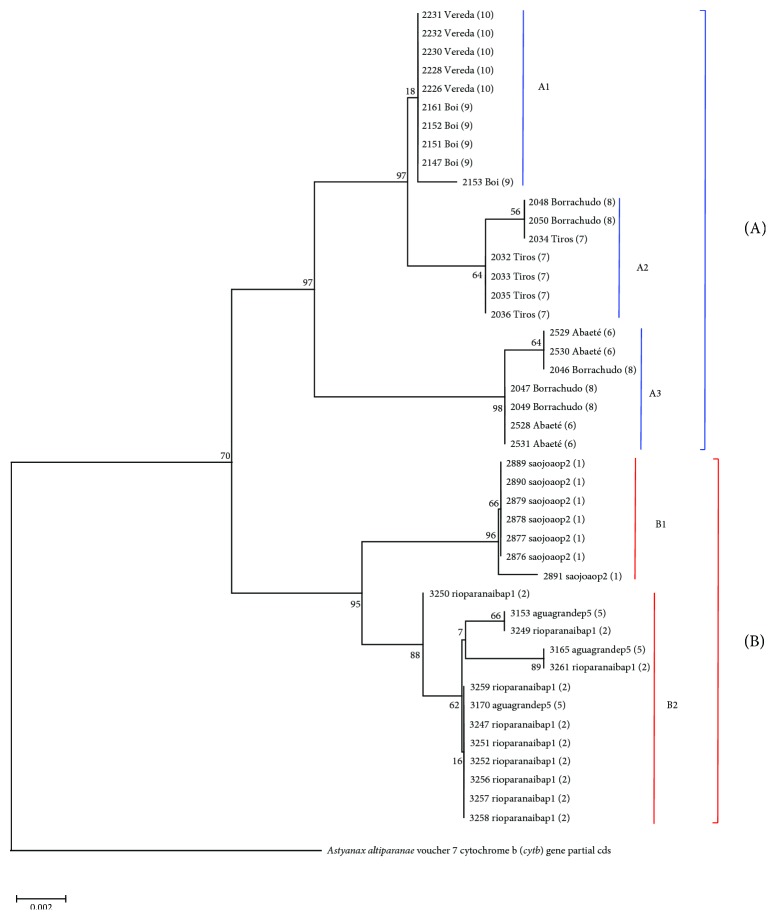
Maximum likelihood phylogram built by MEGA v6.06. The values at each node are the bootstrap for 1000 replications. The numbers are equivalent to the sampling sites of Figure 1:1, São João river; 2, Paranaíba River; 5, Água Grande stream; 6, Abaeté river; 7, Tiros stream; 8, Borrachudo river; 9, Boi river; 10, Vereda Grande stream.

There were two main clades in the phylogram. One of them (clade A) is composed exclusively of* A. rivularis* individuals, while the other (clade B) is composed of* A. paranae*. Clades A and B presented two subclades each. Individuals from Borrachudo river were shared by the two* A. rivularis *subclades. Clade B1 was composed only of individuals from São João River, and clade B2 was composed of the remaining populations from Paranaíba basin (Figure 5).

According to the results found on phylogenetic analysis and haplotype network, each species was separated on two groups from which the genetic distances between and within them were calculated. The groups match exactly with the clades observed on the phylogenetic analysis, so they were named as seen on there. The distances from the species as a whole were also calculated in 0.025. The results are shown in Table 1.

### 3.3. Haplotype Network

**Figure 6:**
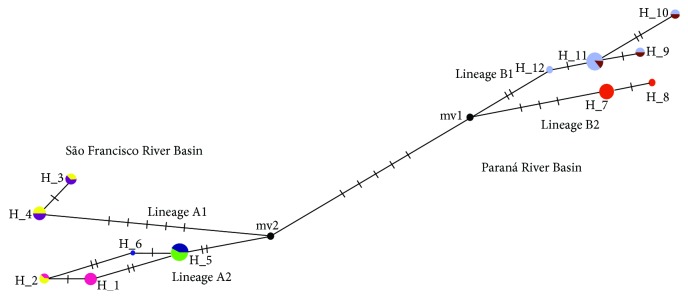
Haplotype network. Each line indicates a mutational event (step). The distance between each node is proportional to the number of mutational steps. The black circles are the missing haplotypes. Sampled populations: 1, Borrachudo River (yellow); 2, Tiros Stream (pink); 3, Vereda Grande stream (light green); 4, Do Boi River (dark blue); 5, Abaeté river (purple); 6, Água Grande stream (brown); 7, Paranaíba river (light blue); 8, São João river (red).

The haplotype network generated by the software system showed two extremities. Each one was from one species, connected at the center by the missing haplotype mv2 (Figure 6). Twelve haplotypes were identified, six from each species, with 22 variable sites of Cytochrome* b *gene. The* A. rivularis*' extremity presented a bifurcation originated from haplotype mv2. Except for haplotype #1, represented by four individuals from Tiros stream, and haplotype #6, represented by only one individual from Do Boi River, the remaining haplotypes were represented by individuals from mixed populations.. In* A. paranae*, São João river specimens were represented by haplotypes #7 and #8, while haplotypes #9, #10, and #11 represent individuals from Paranaíba River and Água Grande stream. The haplotype #12 is present in one individual of Paranaíba River.

## 4. Discussion

The results showed that the species under study have visible morphological differentiation and genetic structuration.* Astyanax paranae* has a fusiform body, compared to* A. rivularis*, with a typical shape of higher water flow environments [25].* A. rivularis* presents lower body depth in addition to a longitudinal elongation when compared to* A. paranae*. Such characteristics are usually associated to environments with lower speed water [25, 26]. According to Atlas das Águas [27], the average water speed at the sampling sites for the Upper Paranaíba populations is higher than that found in the São Francisco River Basin region, which matches with the body form variations found (Figure 4).

These data expand that one by Moreira-Filho and Bertollo [2], who proposed that species of* A. scabripinnis* complex would be morphologically adapted into a wide range of environments. Recent studies reinforce the great morphological plasticity of the genus and demonstrate that different species in the same environment could present distinct phenotypic adaptations [28, 29]. Thus, divergent evolutionary mechanisms at each basin could change the adaptive response, since environmental exploration and interaction with other species affect character selection, habitat colonization, and, hence, genes [30]. This could accelerate the evolutionary process that generates diversification of characters, even driving to allopatric speciation [31], since different genes tend to fix in different populations due to the particular selective pressures of each habitat.

The phylogenetic tree of the local tetras indicates a clear distinction between the two species, with the formation of a clade composed only by* A. rivularis* (clade A) and other composed only by* A. paranae* (clade B). The calculated 2,5% genetic distance corroborates the idea of different species, since April* et al*. [32] established 2% as the threshold of difference on DNA base composition from fish mitochondrial DNA to sustain the separation of distinct species.

In both clades, the relationships found are consistent with the geographic distribution of the rivers in which the individuals were collected. For* A. rivularis* (clade A), the diversification of populations seems to be more recent, due to lower structuration of the populations. Subclade A1 isrepresented by nearby and interconnected watercourses, which explain the clustering observed between them. However, between populations of subclade A2, there is no connection relationship between the rivers. Nevertheless, there is a geographical proximity at some points along the rivers, which would mean that the two initially isolated populations may have some contact, in case of floods, a common occurrence in the region [33], or even due to human intervention. The structuring of populations is more evident among the* A. paranae* (clade B), which demonstrates physical isolation between them. Subclade B1 is composed of an isolated river, which reflects on the structuration observed. Subclade B2 groups two distinct populations, which come from nearby and connected water bodies, since Água Grande stream is a tributary of the Paranaíba River. Despite the geographical proximity between these rivers, unlike the observed in São Francisco River Basin, fauna changes due to floods are improbable, since the hydromorphological characteristics of the basin do not support such event [34].

The haplotype network coincided with the results found in the phylogram of maximum likelihood, with two extremities, each one composed of only one species, which matches the geographic distribution of the basins. Besides the existence of three clades for* A. rivularis* on the phylogram, only two lineages were found for the species. The lineage B corresponds to clades A1 and A2, whose genetic distance is only 0.4%. That fact, in addition to shared mutational steps on same sites, as evidenced in Figure 6, may explain why there are only one lineage for these two different clades. Haplotype mv2 can be seen at the center of the network and could not be collected or may be extinct. According to Coalescent theory [35], mv2 would probably be the ancestral haplotype,* i.e.,* the one that existed before the separation of the basins. Thus, we can infer that the process that drives the establishment and evolution of the ancestral haplotype was different in each basin.

The establishment of* A. rivularis* at the basin probably occurred with the dispersion of two lineages, A1 and A2, from the ancestral haplotype. The presence of individuals from Borrachudo River in both lineages could indicate the occurrence of homoplasy in the sequences, probably due to the geometry of the network [35] or maintenance of the ancestral haplotype [36]. However, in* A. paranae,* firstly there was the establishment of descendent haplotype mv1, derived from ancestor mv2. After this initial event, a secondary dispersion occurred along the basin, which also resulted in two lineages, B1 and B2, from the studied populations (Figure 6). Although lineages within each species appear to be divergent, most notably at* A. rivularis* case, the clustering found in the maximum likelihood phylogram and MANOVA/CVA and the low genetic distances between them indicate that there are only two species involved at the analysis.

The populations of both species, physically separated, may have passed through different evolutionary events, which led to the genetic and morphologic differentiation observed. Reduction or interruption of gene flow among them, due to isolation, leads to the accumulation of unique changes of the evolutionary history of each species. That fact, associated with the observed differentiation along with natural process of genetic drift and natural selection [37], leads us to believe in allopatric speciation as the responsible for the origin of species.

One of the main phenomena that affect this type of speciation is the vicariance [38], which occurred with the* A. rivularis *and* A. paranae* populations. The Upper Paranaíba Arc separates São Francisco River Basin from the Paraná River Basin, since a rock elevation emerged at Meso/Neocretaceous [5]. The rising of the elevation may have led to the isolation of species previously shared by both basins, acting as a vicariant event to them [39]. Different aspects of each basin, i.e., altitude, average speed of water, fauna, and flora composition, may have been agents to the diversification between the two species. Other works on species of the genus suggest the same board of speciation after the uplift of Upper Paranaíba Arc [40].

## 5. Conclusions

Although the identification of these species is often difficult due to the absence of diagnostic morphological characters, the genetic and morphological data shows lines of evidence that populations of* Astyanax rivularis *and* Astyanax paranae* are not intermixed, hence being different evolutionary units. The uplift of the Paranaíba Arc seems to be the main reason for this, reinforcing vicariance and allopatry roles in the evolution of the* Astyanax *genus and the strict relation between natural formation of hydrographic basins and their inhabitant ichthyofauna.

## Figures and Tables

**Table 1:** Genetic distances between clades of *Astyanax rivularis* (A1, A2, and A3) and *Astyanax paranae* (B1 and B2). The values on the diagonal are genetic distances within each clade.

	Clade A1	Clade A2	Clade A3	Clade B1	Clade B2
Clade A1	0.000				
Clade A2	0.004	0.001			
Clade A3	0.011	0.014	0.001		
Clade B1	0.015	0.019	0.020	0.000	
Clade B2	0.021	0.019	0.018	0.010	0.001

## Data Availability

The DNA sequence data used to support the findings on this study have been deposited in the GenBank nucleotide repository under the accession numbers MK756216 to MK756259
